# Cardio-Lipotoxicity of Epicardial Adipose Tissue

**DOI:** 10.3390/biom14111465

**Published:** 2024-11-18

**Authors:** Monica L. Bodenstab, Ron T. Varghese, Gianluca Iacobellis

**Affiliations:** 1 Department of Internal Medicine, Miller School of Medicine, University of Miami, Miami, FL 33136, USA; monica.bodenstab@jhsmiami.org; 2 Sleep—Endocrinology Integrated Clinic, Division of Endocrinology, Diabetes and Metabolism, Department of Medicine, Miller School of Medicine, University of Miami, Miami, FL 33136, USA; rxv492@med.miami.edu; 3 Division of Endocrinology, Diabetes and Metabolism, Department of Medicine, Miller School of Medicine, University of Miami, Miami, FL 33136, USA

**Keywords:** cardiac metabolism, adipokines, obstructive sleep apnea

## Abstract

Epicardial adipose tissue is a unique visceral adipose tissue depot that plays a crucial role in myocardial metabolism. Epicardial adipose tissue is a major source of energy and free fatty acids for the adjacent myocardium. However, under pathological conditions, epicardial fat can affect the heart through the excessive and abnormal influx of lipids. The cardio-lipotoxicity of the epicardial adipose tissue is complex and involves different pathways, such as increased inflammation, the infiltration of lipid intermediates such as diacylglycerol and ceramides, mitochondrial dysfunction, and oxidative stress, ultimately leading to cardiomyocyte dysfunction and coronary artery ischemia. These changes can contribute to the pathogenesis of various cardio-metabolic diseases including atrial fibrillation, coronary artery disease, heart failure, and obstructive sleep apnea. Hence, the role of the cardio-lipotoxicity of epicardial fat and its clinical implications are discussed in this review.

## 1. Introduction

The investigation into epicardial adipose tissue (EAT) began over two decades ago, with pioneering work beginning in the early 2000s by multiple groups, including work from our research group [1]. Since that time, extensive research has been performed on EAT and we now recognize its vast potential as a visceral fat depot that can serve a protective measure to the myocardium via its proximity in homeostatic conditions, and its contribution to pathologic states such as heart failure, coronary artery disease, and atrial fibrillation. For these reasons, an investigation of EAT is paramount as we shift our focus to the importance of organ-specific adiposity.

## 2. What Is It, Where Is It

EAT refers to the deposition of fat tissue that resides between the epicardium and the outer wall of the myocardium. It is uniquely different from its counterpart, pericardial adipose tissue (PAT), both in its blood supply and its biochemical and microscopic makeup. While PAT is a mediastinal fat that resides outside the parietal pericardium, EAT is visceral thoracic fat that abuts the heart itself [2] and shares the same embryogenesis with intra-abdominal fat [3]. PAT receives its circulation from vessels such as the branches of the internal mammary artery, while EAT is supplied by the coronary arteries, sharing its circulatory support with the myocardium [2,4,5]. The importance of EAT comes from this shared microcirculation and the anatomical proximity of EAT to the myocardium, as there is no separation from muscle fascia between the EAT and the myocardium. Given this proximity and shared microcirculation, these two tissues therefore engage in crosstalk. This crosstalk may be in the form of vasocrine signaling or via paracrine cytokine release [6]. Overall, myocardial EAT can primarily be found in the atrioventricular and interventricular grooves as well as over the right ventricular free wall and left ventricular apex. When EAT is found surrounding the coronary arteries, it is deemed pericoronary EAT [2,4]. EAT composes 20% of the heart’s ventricular mass according to autopsy studies, and was slightly higher in women as compared to men [7].

On a microscopic level, EAT is composed of many different cell types, including inflammatory cells, immune cells, nerve cells, stromal cells, and vascular cells; however, it is primarily composed of adipocytes. There is a higher prevalence of preadipocytes compared to mature adipocytes in the EAT, likely due to its rapid metabolism, which results in a significantly smaller overall size of the adipocytes when compared with subcutaneous or peritoneal fat [6]. The adipocytes in EAT are white adipose tissue but are unique in that they have features of brown adipose tissue (BAT) and beige fat [8]. EAT mRNA contains significant amounts of uncoupling protein-1 (UCP-1), a major marker of BAT, in addition to multiple other genes (i.e., PPARy, CD137), which contribute to the induction of white adipose tissue becoming brown-like. Alternately, beige-like cells are those that only express extensive amounts of UCP-1 under stimulation, for example, due to cold exposure or certain drugs [8]. Although not yet well understood, it is purported that EAT may contribute to thermogenesis for the myocardium due to the unique adipocyte functionality of EAT, which may be beneficial in times of hypoxia or ischemia [9]. However, in vivo studies testing this hypothesis of the targeting of UCP-1 in EAT in humans are currently lacking. However, this brown-like feature of EAT is not permanent, and the BAT activity declines with aging, as well as in certain pathologic states. For instance, in advanced CAD, the BAT activity seems to decrease in the setting of downregulation of BAT-related gene expression. To counteract this, an increased expression in pro-inflammatory cytokine genes occurs. In addition to these molecular changes, the sheer quantity of brown adipocytes also decreases in favor of white adipocytes [10,11,12]. Countering the gene expression that reduces BAT-like function of the EAT is a potential therapeutic target for patients with ischemic cardiovascular disease [13].

Under physiologic conditions, EAT is a protective entity. Its transcriptome contains cardioprotective, anti-inflammatory, and anti-atherogenic adipokines such as *ADIPOQ*, coding for adiponectin, and *ADM*, coding for adrenomedullin [5,14]. Additionally, EAT acts as a buffer. Given its continuance with the myocardium, it provides protection against high fatty acid levels, which will be discussed next.

The measurement of epicardial tissue thickness can be performed using a point-of-care handheld portable ultrasound instrument, while the measurement of epicardial fat volume usually requires a CT or MRI scan. Hence, while a EAT thickness with ultrasound measurement may be cost effective, it would require the technical expertise of the user at point of care, while a EAT volume measurement is expensive and also causes exposure to radiation with CT scan imaging.

## 3. Myocardial Metabolism

To understand the impact of EAT on the heart’s physiology and pathophysiology, we must first discuss cardiac metabolism. The myocardium is flexible in its metabolic options, adapting to the availability of different substrates for energy. In steady-state conditions, fatty acids are the main substrate for ATP production. However, others, including ketone bodies, glucose, branched-chain amino acids (BCAAs), and lactate, also serve as sources for cardiac metabolism and cardiac ATP production. The majority, approximately 60–90%, of the heart’s energy comes from fatty acids [15]. This is because free fatty acids are both readily available and yield efficient and high amounts of ATP production for each molecule of FFA. However, cardiac myocytes are unable to produce or store FFAs. Therefore, the cardiac myocytes require a carrier protein to utilize fatty acids, which is known as a fatty-acid-binding protein, moving the FFAs into the cells to undergo subsequent energy production. Of note, other proteins, such as fatty acid translocase (FAT/CD36) or fatty-acid-binding protein 4 (FABP4), also assist in mobilizing FFA into the cells [16]. Once moved across the transporter protein, FFAs are converted into fatty acyl-CoAs. These fatty acyl-CoAs bind to acyl-CoA-binding proteins and enter the mitochondria via the carnitine palmitoyltransferase enzyme. Once inside the mitochondria, the FFAs are degraded via the B-oxidation pathway into the end product of acetyl-CoA to undergo the tricarboxylic acid cycle (TCA cycle or Krebs cycle). During this cycle, NADH and FADH2 yield ATP via the electron transport chain (ETC). Fatty acid oxidation is stimulated by glucagon and sympathetic stimulation from the sympathetic nervous system [17]. When under high energy demand, such as during exercise, fatty acids are also more readily mobilized from the peripheral adipose tissue to supply the heart. Other signaling pathways, such as the inhibition of the rapamycin (mTOR) signaling pathway, also stimulate fatty acid oxidation [18].

The cardiac myocytes also utilize glucose as an energy substrate, but this only accounts for approximately 10–30% the heart’s energy production. To do so, glucose is metabolized either via glycolysis or oxidative phosphorylation. Glycolysis degrades glucose into pyruvate, which can also enter the TCA cycle similarly to the fatty acid pathway. Glucose becomes the favored substrate in the presence of insulin release and therefore during feeding times [19]. The activation of the mTOR pathway also promotes glucose uptake by the heart [20].

In times of stress, BCAAs (branched-chain amino acids) and essential amino acids can be used for energy as well [21]. BCAAs also break down and enter the TCA cycle. However, this energy production is more strictly controlled. Overall, the heart adapts its fuel source based on availability and physiologic conditions to maintain steady energy production regardless of demand. Its energy production is regulated via a complex interplay of hormonal regulation, neural regulation, and intracellular signaling pathways.

## 4. Lipotoxicity of EAT

The fat content in EAT is different from that of subcutaneous tissue, in that EAT has higher saturated fat content and lower unsaturated fat content [22]. When compared to intrabdominal fat, both insulin action [23] and glucose utilization are lower in EAT [23,24].

At the cellular level, ectopic lipid accumulation results in the activation of the unfolded protein response (UPR)/ER stress, oxidative stress, and pro-inflammatory signaling [25,26,27]; the overproduction and accumulation of lipid intermediates such as diacylglycerol and ceramide, that can then induce cell cycle arrest and apoptosis [28,29]; and mitochondrial dysfunction characterized by an impaired capacity for efficient oxidative phosphorylation [30]. Exosome secretion from cardiac myocytes has also been shown to increase through increased extracellular calcium stress or oxidative stress [31,32].

Conditions like ischemia and heart failure induce endoplasmic reticulum dysfunction [33] and thus the misfolding of proteins. These conditions also induce changes in cardiokine secretion, including IL-6, TNF-α (tumor necrosis factor—alpha), follistatin-like 1, adrenomedullin, apelin, and vascular endothelial growth factor (VEGF) [34]. Increased VEGF secretion from cardiomyocytes in type 2 diabetes mellitus increases fatty acid delivery to the heart through autocrine/paracrine signaling and may contribute to cardiovascular disease [35].

The excessive release of fatty acids, leading to intracardiac cell ectopic lipid accumulation, the overexpression of local pro-inflammatory and profibrotic cytokines with pro-arrhythmogenic properties, and increased β-adrenergic receptor activation, results in conditions like heart failure [36]. Adipokines have complex regulatory effects on myocardial fibrosis. Adipokines such as leptin and resistin promote the development of myocardial fibrosis, promote myocardial remodeling and exacerbate cardiac diastolic dysfunction. Other adipokines such as adiponectin, Omentin, apelin, Vaspin, Stamp2 and Nesfatin-1 can inhibit myocardial fibrosis and have a cardioprotective effect [37]. Some myokines, such as myostatin, irisin, brain-derived neurotrophic factor, interleukin-15, fibroblast growth factor-21, and growth differential factor-11, engaged in the regulation of the pathogenesis of HF-related myopathy, can be detected in peripheral blood, and the evaluation of their circulating levels can provide new insights to the course of HF [38].

## 5. Lipo-Cardiotoxicity of EAT

### 5.1. Role of EAT in Cardiovascular Disease

Cardiovascular disease (CVD) remains at the forefront as one of the leading causes of death in the world [39]. Under states of increased fatty infiltration, cardiotoxicity arises, leading to CVD. The causes of cardiotoxicity are multifactorial; however, notably, the disruption of ATP production described previously contributes to the development of CVD. This can occur via oxidative stress, in which excess fatty accumulation can activate the innate immune system and invoke an inflammatory response. Once activated, further pro-inflammatory cytokines are induced, and reactive oxygen species (ROS) and cellular damage occurs. Lipid accumulation therefore can disrupt steady-state myocyte metabolism via mitochondrial damage, impaired substrate uptake, and impaired cellular cycles. Eventually, this can lead to the pathogenesis of cardiovascular disease.

In homeostatic conditions, EAT contributes to lipid regulation [40]. In pathological states, such as obesity, EAT itself can also negatively impact myocardial metabolism. In studies of animals fed high-fat diets, as well as obese humans, the quantity of EAT exceeds those of healthier subjects. Interestingly, in those with more visceral adiposity, the disparity is even greater [24,41]. EAT is also further affected by a poorly balanced diet and is affected to a greater extent by dietary changes than its counterpart PAT. In high-fat diets, there is a higher proportion of fatty acid storage, thought to be due to a disproportionate oxidation of non-esterified fatty acids [42]. While some of the excess lipid can be neutralized and stored as triglycerides, excess can unfortunately lead to apoptosis in the cardiac cells. The free fatty acid oxidation capacity becomes saturated, leading to a buildup of fatty acid intermediates that remain in the cytoplasmic regions and result in further cardiac damage.

On a molecular level, this fat accumulation is spurred by EAT dysregulation in pathologic states. Excess FFAs, recognized as endogenous antigens, activate toll-like receptors (TLRs) in epicardial macrophages and adipocytes alter cellular signaling to upregulate transcription factor expression. These transcription factors, notably NF-k Band FOS, lead to the overexpression of pro-inflammatory factors, including IL-1, IL-6, IL-8, and TNF-a [43]. As a result of this altered activation, macrophages from the *transdifferentiated* adipocytes lead to the downstream upregulation of intracellular adhesion molecule-1 (ICAM-1), MCP-1, and, again, IL-6. As a result of this, lipid accumulation occurs, leading to an atherosclerotic plaque.

It is important to note that there is altered gene expression in pathologic states, such as in diabetes mellitus, where certain proteins of lipid metabolism in the EAT are upregulated. These proteins include lipase G (LIPG), solute carrier family 7 member 5 (SLC7A5), and solute carrier family 16 member 10 (SLC16A10) [44]. This is important in light of some studies showing that the diabetic EAT transcriptome is significantly different when compared to diabetic SAT, and is highly enriched with genes involved in innate immune responses and endothelium, like Pentraxin3 (PTX3) and endothelial lipase G (LIPG) [44]. The differences in transcript between subcutaneous and EAT depots, and in patients with and without diabetes, warrants further studies for mechanistic insights.

Overall, the myocardium is minimally capable of fat storage; therefore, under states of high lipid levels, the heart undergoes dysfunction, which can lead to pathologic states and heart failure. This is especially true in morbidities such as morbid obesity and diabetes.

**Figure 1:**
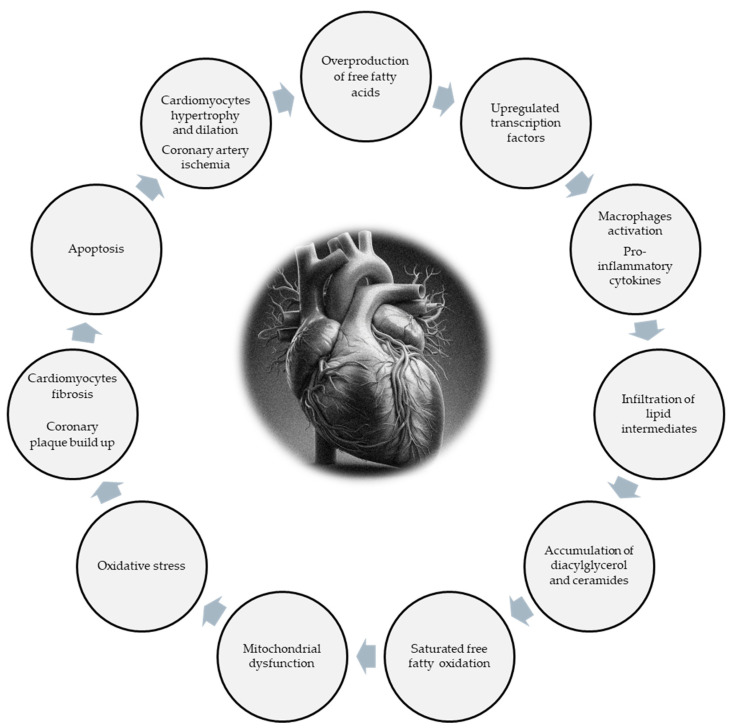
Cardio-lipotoxicity of epicardial adipose tissue. This graphic summarizes the mechanisms underlying the cardio-lipotoxicity of epicardial adipose tissue. The cascade of events hereby described may follow alternate or preferred pathways due to the concurrence of exogenous conditions such as diabetes, obesity, coronary artery disease, or atrial fibrillation. The excessive production and release of free fatty acids through paracrine and vasocrine pathways from epicardial fat into the adjacent myocardium and coronary artery leads to an upregulation of endogenous transcription factors that activate M1 macrophages and increase the production of pro-inflammatory adipokines. The increased inflammatory milieu causes the accumulation and infiltration of lipid intermediates such as diacylglycerol and ceramides within the cardiac cells and coronary lumen that contribute to mitochondrial dysfunction and oxidative stress. The excessive lipid accumulation, due to the saturated capacity to oxidize and utilize the ectopic lipid infiltration, causes coronary plaque build-up and cardiomyocyte fibrosis. Apoptotic changes will ultimately lead to cardiac hypertrophy and/or dilation and coronary artery ischemia.

Although cardio-lipotoxic mechanisms linking EAT to cardiovascular diseases are complex and not completely understood yet, a pathway of events seems to be plausible, as depicted and summarized in Figure 1. The excessive production and release of free fatty acids through paracrine and vasocrine pathways from epicardial fat into the adjacent myocardium and coronary artery leads to an upregulation of endogenous transcription factors that activates M1 macrophages and increases the production of pro-inflammatory adipokines. The increased inflammation causes the accumulation and infiltration of lipid intermediates such as diacylglycerol and ceramides within the cardiac cells and coronary lumen that contribute to mitochondrial dysfunction and oxidative stress. The excessive lipid accumulation, due to the saturated capacity to oxidize and utilize the ectopic lipids, causes coronary plaque build-up and cardiomyocyte fibrosis. Apoptotic changes will ultimately lead to cardiac hypertrophy and/or dilation and coronary artery ischemia.

### 5.2. Role of EAT in Atrial Fibrillation

Atrial fibrillation (AF) is the most common serious arrhythmia, requiring clinical management. AF can be classified as either paroxysmal, persistent, long-standing persistent, or permanent [45]. After cardioversion or antiarrhythmic attempts, there is still a recurrence risk of 40–50%, and unfortunately AF leads to an increased risk of stroke, heart failure, peripheral embolism, and death [46]. There are many contributing factors postulated to play a role in AF development and recurrence, including EAT. Mechanisms for EAT contribution may include fatty infiltration, fibrosis, inflammation, oxidative stress, and altered gene expression, to name a few; although, other possible mechanisms are also suggested in the literature [47].

#### 5.2.1. Inflammation

It is well-indicated that AF is concomitant with inflammation [48], which may be predicted by laboratory evaluations such as hs-CRP [49]. Under pathologic conditions, EAT can act as a pro-inflammatory as well as a pro-fibrotic tissue. Pro-inflammatory markers, including TNF-a, and various interleukins, including IL-1B, IL-6, and IL-8, are secreted by EAT and may facilitate arrhythmogenic activity [47]. EAT has been shown to have a higher inflammatory state in patients with AF, which was identified via a heightened glucose uptake on PET/CT and was unrelated to BMI. The EAT inflammatory activity has also been found to be greater than in the pericardial subcutaneous tissue [50].

Cardiac lipotoxicity may provoke oxidative stress, DNA damage, inflammation, and insulin intolerance, resulting in morphological changes and the cellular dysfunction of atria [51,52]. Various changes in atria including cardiac hypertrophy, fibrosis, gap junction remodeling, and myocardial injury are thought to provide risk to develop atrial fibrillation [53,54,55,56].

While obesity contributes to a pro-inflammatory state and is a well-known risk factor for AF [57], the association between AF and cardiac adipose tissue has been found to be independent of obesity [58]. The accumulation of site-specific fat seems more influential than overall body fat in increasing overall cardiovascular risk [36].

#### 5.2.2. Fibrosis

Pro-fibrotic cytokines including matrix metalloproteinases (MMPs), transforming growth factors (TFGs), and activin A are also released from EAT. Given EAT’s unique anatomical location and contiguity with the atrial myocardium, these substances can diffuse directly, which can contribute to AF, as fibrosis is known to have a pathogenic role in the development of AF. While MMPs regulate the extracellular matrix physiologically, their overexpression can lead to fibrosis [59]. It has been shown in rat atria that TGFB1 and 2 are upregulated in EAT-conditioned medium. These factors promote fibrosis and are further mediated by secretin of activin A, also from the EAT [59].

#### 5.2.3. Fatty Acid Infiltration

The physical disruption of cardiomyocytes due to free fatty acid infiltration from the EAT can lead to electromechanical changes in the atrium as well. EAT has been shown to infiltrate the adjacent myocardium histologically [60]. This infiltration is postulated to lead to the disorganization of the myocardium through cardiomyocyte separation [61], which leads to conduction slowing, delay and re-entry. The role of PAT volume was initially recognized as a directly corresponding predictor of AF.

#### 5.2.4. Relation of EAT Thickness

Previous recognition of the role of PAT volume corresponding to an independent predictive risk factor for AF has been noted in multiple studies, independent of other adiposity measures and LA enlargement [62,63,64,65]. However, more recent studies elucidate the purported connection between EAT volume and AF severity and persistence. EAT thickness seems to relate to the persistence of AF, as individuals with chronic AF were found to have significantly increased epicardial fat thickness when compared with individuals with paroxysmal AF [66]. This was found to be independent of other AF risk factors [47,64,67,68]. EAT thickness is also associated with atrial conduction delays that are notable on an electrocardiogram [69].

### 5.3. Coronary Artery Disease

CAD is a type of atherosclerosis which results from coronary artery occlusion and can lead to severe cardiovascular disease in the form of myocardial infarction or heart failure due to tissue hypoxia. The risk of atherosclerosis is enhanced by different risk factors such as diabetes, hypertension, and obesity. However, increased visceral adiposity specifically is linked to increased risk [70]. Atherosclerosis is well known to be a disease of cholesterol storage; however, more recently, the chronic inflammatory component of the disease process is more widely recognized [71]. EAT also has a role in contributing to CAD, as it can contribute to this inflammation and immune response among various other mechanisms. EAT is thought to contribute to the pathogenesis of CAD, primarily via inflammatory mechanisms with both adaptive and innate immune responses, and the result of a pro-atherogenic transcriptome.

The mechanisms underlying lipotoxicity resulting in endothelial dysfunction include oxidative stress, inflammation, mitochondrial dysfunction, and endoplasmic reticulum (ER) stress, as well as cell death. Treatment with exercise, diet, or pharmacologic agents directly or indirectly reduces lipids in the plasma to ameliorate endothelial dysfunction in coronary artery disease [72].

EAT in CAD states has a greater level of inflammation compared to both subcutaneous adipose tissue and other visceral fat depots. Pro-inflammatory markers in the EAT are found in the form of macrophages, cytokines, and various other immune cells [73,74]. Imbalance between pro-inflammatory and anti-inflammatory adipokine secretion also affects CAD progression and the altered transcriptome of EAT favors pro-atherogenesis, leading to altered adipokine production [40,73,74]. CAD individuals have a lower expression of adiponectin, an anti-inflammatory cytokine, and higher expression levels of alternate pro-inflammatory adipokines [5].

This pro-atherogenic transcriptome in CAD is suggested by the upregulation of pro-inflammatory cytokine genes. These include cytokines such as tumor necrosis factor (TNF), IL-6, CCL2, and other adipokines (chemerin, intelectin 1, resistin, serglycn) that are pro-inflammatory [43,75,76]. Resistin, for example, regulates the endothelial function and is related to the increased permeability of the endothelial cells [77]. Mediators of the innate response in EAT are activated and contribute to the upregulation and expression of these inflammatory cytokines. These include JUN N-terminal kinase (JNK), nuclear factor-kB (NF-kB), and toll-like receptors [74].

Other pro-inflammatory findings include the presence of mast cells and dense macrophage infiltration in the EAT [78]. Again, disequilibrium is present in patients with CAD, as the pro-inflammatory M1 macrophages are found in abundance compared with anti-inflammatory M2 macrophages [78]. This macrophage infiltration is noted by increased CD45 levels in the EAT, which are a marker of hematopoietic cells, when compared to other omental fat depots [74]. The adaptive immune response also plays a role in EAT inflammation, as high levels of CD8+ T cells have been found in CAD states [78].

Oxidative stress leading to endothelial cell damage is another driver of coronary atherosclerosis.

Specifically in patients with diabetes, there is upregulation in the EAT of advanced glycation end products (AGEs) and their receptors (RAGEs). This increased binding contributes to oxidative stress [44].

Finally, the EAT likely contributes to the fatty infiltration of the coronary arteries given its own dense fat content, and via increasing fatty acid uptake. Fatty acid influx into the coronary arteries from the EAT is mediated by enzymes such as fatty-acid-binding protein 4 (FABP4) and by group II secretory phospholipase A2 (sPLA2-II), which could contribute to the plaque formation and accumulation [79,80]. sPLA2-II is the rate-limiting enzyme of pro-inflammatory lipid synthesis and has been found in higher concentrations within the EAT in patients with CAD [80]. Further findings in patients with both CAD and type 2 diabetes have shown an upregulation of lipid metabolism and nutrient transport genes in the EAT, such as the proteins endothelial lipase, solute carrier family 7 member 5 (SLC7A5), solute carrier family 16 member 10 (SLC16A10), and lipase G (LIPG) [81]. This upregulation is increased when compared to patients with CAD alone [44]. Thus, further studies into the differences in gene expression in EAT in subjects with Type 2 diabetes mellitus may shed light into the role of EAT dysfunction in pathogenesis of coronary artery disease in diabetes mellitus.

There is a high concentration of lipids in the EAT of patients with diabetes, including unsaturated fatty acids (12:0 and 16:0), sphingolipids (SPA, S1P, C14-Cer, C16-Cer, C18:1-Cer, C18-Cer, and C24:1-Cer), and ceramides [82,83]. Trans-fatty acids and conjugated fatty acids are also increased. This increased excessive EAT fat likely contributes to the cardiomyopathy noted in patients with diabetes as well.

### 5.4. Heart Failure

Heart failure is a broad term that includes abnormalities in multiple components of the heart and can be further divided into heart failure with reduced ejection fraction (HFrEF), where systolic function is reduced and ejection fraction is <40%, and heart failure with preserved ejection fraction (HFpEF), in which the diastolic function of the heart is impaired. A link between EAT and the pathogenesis of HFpEF is likely, with multiple mechanisms suggested in the literature.

Firstly, a correlation between EAT volume or thickness and HFpEF has been established in multiple studies, although potential confounding variables such as other cardiovascular diseases, diabetes, or obesity were not necessarily ruled out. Regardless, an assessment of volume or thickness via multiple imaging modalities (e.g., ultrasound, cardiac MRI) found that patients with HFpEF had a significantly higher quantification of EAT. The correlation between EAT thickness and HFrEF is less understood and more unpredictable, as the EAT burden can be either higher or lower than in control individuals. These findings could be attributed to the differing metabolic demands of HFrEF patients or the variability in ventricular remodeling as well [84,85,86,87,88,89]. However, a large and fibrotic EAT can cause mechanical effects on the heart, which may contribute to both systolic and diastolic dysfunction [90]. Additionally, given the direct effacement between the EAT and the myocardium, cardiomyocyte disarray, and eventual dysfunction and apoptosis, can occur from excessive EAT-derived fatty acids that then infiltrate the myocardium. The levels of intramyocardial fat correlate with the left ventricular diastolic dysfunction found in patients with HFpEF. Interestingly, these myocardial fat levels are significantly higher than in patients with HFrEF or in control individuals [36,42,90,91,92].

The unique proteome of epicardial adipose tissue found in heart failure subjects has also been identified as a potential cause of both the diastolic and *systolic* function of the heart. In HF subjects, there are increased pro-inflammatory and pro-fibrotic cytokines present. Via its paracrine and endocrine function, EAT upregulates factors such as a1-antichymotrypsin (ACT; also known as serpinA3), MMP14, and creatine kinase B-type in patients with heart failure [92]. The EAT in heart failure patients also shows an increased mRNA expression of the tumor suppressor gene, and the inflammatory marker TP53. Levels are markedly higher than in the subcutaneous fat alone of those subjects [93]. TP53 levels inversely correlate with adiponectin levels (anti-inflammatory) in those with heart failure [94].

Finally, neurohormonal mechanisms from the EAT are also implicated in the pathogenesis of HF. EAT in steady states is a source of noradrenaline and adrenaline, the two foundational catecholamines of the sympathetic nervous system. However, in HF states, these catecholamines are upregulated. Noradrenaline levels in the EAT compared to plasma were two-fold higher. More notably, levels in the EAT versus subcutaneous fat were found at a 5.6-fold increased level [95]. Tyrosine hydroxylase and dopamine B-hydroxylase, the biosynthetic enzymes of catecholamines, are also upregulated in the EAT when compared with the subcutaneous fat in those same patients [95]. In HFrEF patients, it could be said that the increased catecholamine activity may lead to worsening of the systolic function.

Studies show that EAT thickness is increased in HFpEF patients compared to those with HFrEF, and propose that epicardial fat is associated with smaller cardiac chamber sizes in HFpEF, suggesting that epicardial fat acts as a constraint to cardiac dilation [96,97].

### 5.5. Obstructive Sleep Apnea

Obstructive sleep apnea (OSA) and epicardial fat are both associated with the development of type 2 diabetes mellitus and metabolic syndrome, and metabolic syndrome is more likely to occur in those with OSA [98].

Studies have also noted that the EAT thickness, measured using echocardiography, is higher in people with OSA than those without, and further noted that increasing EAT thickness corelates with severity of OSA [99]. In patients with metabolic syndrome, upper body fat deposition index and epicardial fat showed the best association with OSA [100]. Studies also show that adipocyte size does not correlate with EAT thickness, suggesting that EAT deposition associated with obesity is not caused by adipocyte hypertrophy [101]. Even in patients with OSA who are not obese, EAT thickness was higher when compared to controls, and the oxygen desaturation index was a significant predictor of EAT thickness [102]. The association of EAT with OSA, both cardiovascular risk factors, is independent of obesity, and changes in EAT could explain up to 38% of variance in severity of OSA [103].

Other studies also note that EAT volume as measured by CT scan also correlate with OSA severity [104].

Intermittent hypoxia followed by rapid reoxygenation in OSA leads to formation of reactive oxygen species, and this is a key factor in pathogenesis of OSA [105]. At the level of adipose tissue, this leads to change in macrophage population to a predominantly pro-inflammatory M1 macrophage population. This subsequently leads to the formation of pro-inflammatory cytokines and adipokines, including TNF alpha and IL-6, worsening OSA [106,107].

Various mechanisms also contribute to lipotoxicity in OSA, including activated lipolysis in the adipose tissue, decreased lipid clearance from the circulation, and accelerated de novo lipid synthesis. The oxidization of atherogenic lipoproteins, adipose tissue dysfunction, hormonal changes, and the reduced function of HDL particles in OSA also play a role in the lipotoxicity [108].

Studies on the fractional clearance rate of lipids in subjects with OSA have shown that severe OSA decreased the lipolysis of triglyceride-rich lipoproteins and delayed the removal of remnants, and that CPAP treatment may be effective to restore the lipolysis rates [109].

While use of Continuous Positive Airway Pressure for 8–12 weeks has not been shown to improve visceral or subcutaneous fat [110,111]. However, the recent SURMOUNT OSA trial showed the key role of weight loss with tirzepatide, a dual glucagon like peptide 1—(GLP-1) glucose-dependent insulinotropic polypeptide (GIP) analogs showed clinically significant reductions in body weight, the severity of OSA (as measured by apnea hypopnea index), blood pressure, and inflammation with GLP-1A therapy [112,113]. Liraglutide has also been shown to reduce AHI, blood pressure, HbA1c, and weight in OSA [114]; thus, the benefit from GLP-1 seems to be a class effect. The American Thoracic Society has also called for individually tailored interventions to tackle weight loss as a critical intervention in treatment of OSA [115].

Interventions like bariatric surgery have shown a significant reduction in epicardial fat volume as early as six months after bariatric surgery; however, the loss of epicardial fat volume was less in subjects with OSA [116]. Omental adipose tissue sampling in subjects undergoing bariatric surgery did not show an association of OSA with total macrophage infiltration [117].

## 6. Discussion: Summary and Future Directions

Epicardial adipose tissue plays a key role in the pathogenesis of multiple cardiovascular diseases, such as atrial fibrillation, coronary artery disease, heart failure, and obstructive sleep apnea. Although complex and not completely understood yet, the lipotoxic effects of pathological EAT on the proximal myocardium and coronary arteries are considered important mechanisms and substrates for the development and progression of those conditions.

The accessibility regarding the measurement of this visceral fat depot using noninvasive methods for measuring EAT thickness, volume and density can help the stratification of the cardiovascular risk. Furthermore, studies also show the strong correlation between EAT thickness (using ultrasound—echocardiogram) and EAT volume (by CT imaging) [118].

Lifestyle changes, including caloric restriction and exercise for as little as 6 weeks, has been shown to reduce epicardial fat volume [119,120]. Both the high-intensity interval training (HIIT) and medium-intensity continuous training (MICT) formats of exercise have been shown to reduce EAT and PAT, as early as 2 weeks in, in subjects with both normal and impaired glucose tolerance [121]. Similarly, PCSK9 inhibitors have also been shown to reduce EAT thickness after 6 months of therapy [122].

In addition, EAT can serve as a target for drugs such as GLP-1As, dual GLP1-GIPAs, and sodium glucose transporter2 inhibitors (SGLT2is), modulating the fat and the lipids. Treatment with various medications including Metformin [123,124], Dapagliflozin [123], Liraglutide [125], Pioglitazone [126], Semaglutide [127], and Dulaglutide [127] have been shown to result in a reduction in the EAT volume in subjects with obesity. The specific role of these medications, having impacted cardiovascular conditions by modulating EAT, is an area that requires further work and attention. Statins, specifically Atorvastatin, has been shown to reduce EAT thickness [128,129,130,131].

Bariatric surgery has been shown to reduce EAT volume by 27% as early as 6 months following surgery; however, patients with OSA had less EAT volume loss after bariatric surgery [116].

This key work has transformed our understanding of the role of EAT in cardiovascular diseases. Further investigations focused on the role and implications of EAT cardio-lipid metabolism are certainly warranted.

## Data Availability

Not applicable.

## Abbreviations

Epicardial adipose tissue (EAT), pericardial adipose tissue (PAT), glucagon-like peptide-1 (GLP-1), sodium glucose transporter2 inhibitors (SGLT2is), obstructive sleep apnea (OSA), Continuous Positive Airway Pressure (CPAP), IL (Interleukin).
